# Separation and purification of antioxidant peptides from *Idesia polycarpa* Maxim. cake meal and study of conformational relationship between them

**DOI:** 10.1002/fsn3.4325

**Published:** 2024-07-10

**Authors:** Lei Dou, Zimu Zhang, Wenqing Yang, Yaobing Chen, Kai Luo, Jianquan Kan

**Affiliations:** ^1^ College of Biological and Food Engineering Hubei Minzu University Enshi China; ^2^ College of Food Science Southwest University Chongqing China

**Keywords:** *Idesia polycarpa* Maxim. cake meal, molecular docking, network pharmacology, quantum chemistry, separation and purification

## Abstract

In this study, peptides were isolated and purified from *Idesia polycarpa* Maxim. cake meal for the first time, with the aim of discovering peptides with excellent antioxidant properties. Peptides were isolated and purified from the cake meal using ultrafiltration and dextran gel chromatography. Fractions with significant antioxidant activity were identified by mass spectrometry (MS) and the peptides were screened and characterized using techniques, such as network pharmacology and molecular docking. The results showed that the CIPs‐I‐F2 fractions possessed excellent antioxidant activities, and a total of seven peptides were screened, with the main targets of action including serine/threonine‐protein kinase AKT (AKT1), signal transducer and activator of transcription 3 (STAT3), and matrix metalloproteinase 9 (MMP9), among which ISKPTWADF had the highest total binding energy to the target. ISKPTWADF was synthesized in vitro by solid‐phase synthesis and showed a dose‐dependent protective effect against the hydrogen peroxide (H_2_O_2_)‐induced oxidative damage model in human hepatocellular carcinoma HepG2 cells, with its main active site on the tryptophan indole ring at position 52N‐127H.

## Graphical abstract

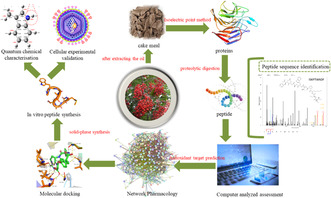

## 1 INTRODUCTION

Generally, during cellular energy metabolism under aerobic conditions, a portion of the oxygen in the electron transport chain is reduced to generate a series of biologically active oxygen‐containing molecules known as reactive oxygen species (ROS), which fulfill crucial roles in cell signaling, immune responses, and various physiological processes (Nathan & Cunningham‐Bussel, 2013). Oxidative stress occurs when the production of reactive oxygen species deviates from normal or surpasses the cell's scavenging capacity (Wang et al., 2022). Research indicates that the metabolic reaction of cytochrome P450 to xenobiotics induced by exposure to toxic compounds serves as a significant ROS‐generating pathway upon invasion by xenobiotics into the cell (He et al., 2017). Cellular damage resulting from oxidative stress typically correlates with the overproduction of free radicals, which are highly reactive molecules capable of inducing oxidative stress within the cell, thereby precipitating various cytopathic alterations, including DNA damage and protein denaturation (Poprac et al., 2017).

Targeted drug studies aimed at mitigating free radical generation and its associated signaling pathways have emerged as a pivotal strategy in antioxidant research, with particular emphasis on certain natural products. For instance, quercetin can regulate mitochondrial function, apoptosis, and autophagy via the nuclear factor erythroid 2‐related factor 2 (Nrf2) signaling pathway, thereby modulating antioxidant and anti‐inflammatory responses to safeguard cells against oxidative damage and inflammation‐induced injury (Zhang et al., 2024). Similarly, Eriodictyol exhibits the potential to ameliorate nonalcoholic fatty liver disease (NAFLD) by modulating autophagy mediated by ubiquitin A‐52 (UBA52) and upregulating the Nrf2/HO‐1 (heme oxygenase‐1) signaling pathway to attenuate oxidative stress (Cai et al., 2024). Antioxidant research should also focus on antioxidant enzymes possessing free radical scavenging functions. Reduced expression and activity of these enzymes leads to increased intracellular oxidative stress, thereby contributing to disease pathogenesis (Kaur et al., 2023). Upon invasion by the yellow fever virus, cells experience heightened ROS production alongside a notable reduction in the activity of antioxidant enzymes, such as superoxide dismutase (SOD) and glutathione peroxidase (GPx). However, the detrimental effects of oxidative stress can be efficiently alleviated through the modulation of antioxidant enzyme expression and activity (Coelho Ferraz et al., 2024). In future antioxidant research, emphasis should be placed on the targeted regulation of free radicals and their associated signaling pathways, alongside the modulation of antioxidant enzyme expression and activity.

*Idesia polycarpa* Maxim., a member of the genus *Idesia* within the Flacourtiaceae family, is esteemed for its substantial nutritional value due to its fruits being abundant in proteins, vitamins, polyphenols, and various other essential nutrients. Studies have demonstrated that an ethyl acetate extract derived from *I. polycarpa* Maxim. fruit can mitigate body weight gain, decrease secretion of inflammatory cytokines, enhance glucose tolerance and insulin sensitivity, and alleviate hepatic steatosis in mice subjected to a high‐fat diet (Huang et al., 2023). The fruit of *I. polycarpa* Maxim., a type of woody oilseed plant, contains up to 43.6% oil and is rich in linoleic acid, linolenic acid, and other unsaturated fatty acids. It serves as an ideal raw material for the industrial preparation of conjugated linoleic acid (Li et al., 2019). *Idesia polycarpa* Maxim. boasts a comprehensive array of amino acids, offering high nutritional value and serving as a premium source of novel vegetable oils (Zhang et al., 2020). To date, the exploitation of *I. polycarpa* Maxim. has primarily concentrated on oil processing and refining, leaving the oil‐derived byproduct, *I. polycarpa* Maxim. cake meal, underutilized. Given its valuable protein content and richness in various amino acids and minerals, *I. polycarpa* Maxim. cake meal holds significant potential as a nutritional supplement for both humans and animals (Yang et al., 2023). Antioxidant peptides have garnered considerable interest due to their remarkable efficacy in showcasing antioxidant activity across human and nonhuman cellular models. These peptides primarily diminish ROS generation by impeding oxidative stress, while concurrently enhancing the synthesis and performance of endogenous enzymes, thereby augmenting cellular antioxidant capacity. Therefore, the quest for antioxidant peptides within the *I. polycarpa* Maxim. cake meal holds substantial practical significance.

Network pharmacology, an emerging interdisciplinary field at the intersection of systems biology and pharmacology, concentrates on the design of multitarget drug molecules by scrutinizing biological system networks. The integration of network pharmacology and molecular docking techniques enables a more precise elucidation of the binding modes and mechanisms of action between drugs and targets. Network pharmacology identified tyrosine‐protein kinase SRC (SRC), MAP kinase extracellular signal‐regulated kinase 2 (ERK2) (MAPK1), transcription factor AP1 (Activator protein 1) (JUN), and signal transducer and activator of transcription 3 (STAT3) as the core targets for *Moringa oleifera* leaf antiosteoporotic peptides, while molecular docking identified DPYLGK as a representative active peptide of *Moringa oleifera* leaf proteins (Men et al., 2024). Moreover, quantum chemical techniques facilitate the exploration of peptide conformational relationships and active sites. For instance, quantum chemistry pinpointed the active site of the watermelon seed antioxidant peptide (P1) to reside at the CH group of Arginine (Wen et al., 2021). Furthermore, quantum chemistry enables simulation of peptide conformational changes and interactions with biomolecules, elucidating their stability and antioxidant properties.

In this study, peptides from *I. polycarpa* Maxim. cake meal were isolated and purified using ultrafiltration and dextran gel chromatography to identify the fraction exhibiting the highest antioxidant activity. Mass spectrometry (MS) was utilized to characterize this active fraction, ensuring the accuracy of its chemical structure and sequence. Based on these findings, the key targets of antioxidant action and peptides with optimal bioactivity and safety were further screened using network pharmacology and molecular docking techniques and characterized utilizing quantum chemical methods.

## 2 MATERIALS AND METHODS

### 2.1 Materials

*Idesia polycarpa* Maxim. cake meal was obtained from laboratory oil extraction (degreased using hexane).

Complex protease (120 μ/mg, mainly composed of protein endonucleases, exonucleases, and flavor enzymes), 1,1‐diphenyl‐2‐picrylhydrazyl (DPPH), and 2,2‐azino‐bis (3‐ethylbenzothiazole‐6‐sulfonic acid) diammonium salt (ABTS) were purchased from Shanghai Yuanye Bio‐Technology Co., Ltd. (Shanghai, China). Human hepatocellular carcinoma HepG2 cells were provided by Hubei Provincial Key Laboratory of Rheumatic Disease Generation and Intervention (Hubei, China). Enhanced Cell Counting Kit‐8 (CCK‐8) by Shanghai Beyotime Biotech Inc. and all other chemicals are of HPLC (high‐performance liquid chromatography) or analytical grade.

### 2.2 Preparation of peptides

Based on previous laboratory studies, *I. polycarpa* Maxim. cake meal protein was prepared using alkali extraction and acid precipitation under specific conditions: a reaction temperature of 55°C, a reaction time of 2 h, a material‐to‐liquid ratio of 20:1, and a system pH of 10 (Yang et al., 2023). The protein was then subjected to enzyme digestion with a complex protease, which involved a digestion time of 2.7 h, a digestion temperature of 45.8°C, a pH of 8, and the enzyme‐to‐substrate ratio was 29:1 (mg:g).

### 2.3 Separation and purification

The proteolytic digest underwent ultrafiltration through a 0.22‐μm membrane, followed by sequential separation and purification using ultrafiltration centrifuge tubes with molecular weight (MW) cut‐offs of 10 and 3 kDa. This process resulted in the successful separation of the digest into three fractions based on different molecular weights: CIPs‐I fraction (MW < 3 kDa), CIPs‐II fraction (MW 3–10 kDa), and CIPs‐III fraction (MW > 3 kDa).

The separation and purification process involved the utilization of a Sephadex G‐25 gel chromatography column, pre‐equilibrated with ultrapure water, flowing at a rate of 0.6 mL/min within a Sephadex G‐25 column (1.6 × 80 cm). Following equilibration, 2 mL of the sample solution was introduced to the pre‐equilibrated Sephadex G‐25 column, with subsequent collection of distinct fractions at a flow rate of 0.6 mL/min, and measurement of sample absorbance at 280 nm.

### 2.4 Determination of secondary structure by FTIR method

The secondary structure of the ultrafiltration components was analyzed using Fourier infrared spectroscopy. The ultrafiltration components were mixed with potassium bromide (KBr), pressed into films, and then scanned using a Fourier infrared spectrometer. The spectral bands were scanned within a range of 4000–400 cm^−1^, with a resolution of 4 cm^−1^, and a total of 64 scans were performed. Subsequently, the spectra were deconvolved using Omnic software, and the results were further processed through smoothing and analyzed using PeakFit v4.12 software, specifically employing second‐order derivative analysis.

### 2.5 Antioxidant activity analysis

#### 2.5.1 DPPH radical scavenging activity

The DPPH radical scavenging activity was determined, based on the method proposed by Wang et al. (2021) and further improved upon it. Various concentrations of sample solutions (0.1, 0.15, 0.2, 0.25, 0.3, 0.35, and 0.4 mg/mL) were prepared and combined with an equal volume of 0.1 mmol/L DPPH solution. The resulting mixture was allowed to react for 30 min at room temperature in darkness, and the absorbance was measured at 517 nm. Glutathione (GSH) served as the positive control. The DPPH radical scavenging activity was determined using the following equation:

$$\begin{array}{c} \text{DPPH radical scavenging activity}\;\left(\%\right)=\left(1-\frac{A_{\text{sample}}-A_{\text{control}}}{\;A_{\text{blank}}}\right)\\ \;\times 100\% \end{array}$$

where A_blank_ is the absorbance of the DPPH–ethanol; A_sample_ is the absorbance of the sample with DPPH; and A_control_ is the absorbance of the sample without DPPH.

#### 2.5.2 ABTS radical scavenging activity

The ABTS radical scavenging activity is determined building upon the methodology developed by Zhang et al. (2023). Initially, ABTS (7 mM) and potassium persulfate (K_2_S_2_O_8_) (2.45 mM) solutions were combined in equal proportions and left in darkness for 16 h to generate an ABTS stock solution. Subsequently, this solution was diluted to achieve an absorbance of 0.7 ± 0.02 at 734 nm. Samples of various concentrations (0.2, 0.4, 0.6, 0.8, and 1.0 mg/mL) were dispensed in 0.2 mL aliquots, and 4 mL of each sample was mixed with the ABTS dilution. The resulting mixture was allowed to react at room temperature for 6 min, followed by measurement of the absorbance at 734 nm. GSH was used for positive control. The percentage clearance effect was expressed as:

$$\begin{array}{c} \text{ABTS radical scavenging activity}\;\left(\%\right)=\left(1-\frac{A_{\text{sample}}-A_{\text{control}}}{\;A_{\text{blank}}}\right)\\ \;\times 100\% \end{array}$$

where A_blank_ is water, instead of sample; A_sample_ is the absorbance of the sample with ABTS; and A_control_ is the absorbance of the sample without ABTS.

#### 2.5.3 Hydroxyl radical scavenging activity

The hydroxyl radical scavenging activity was determined, following the methodology outlined by Ren et al. (2023), with enhancements. Initially, various peptide components were dissolved to prepare sample solutions with concentrations ranging from 2 to 10 mg/mL. Subsequently, 0.5 mL of each sample solution was sequentially mixed with equal volumes of 9 mM ferrous sulfate (FeSO_4_), 9 mM salicylic acid, and 9 mM hydrogen peroxide (H_2_O_2_), followed by incubation at 37°C for 30 min. After centrifugation at 6000 rpm (revolutions per minute) for 10 min, the supernatant was collected, and the absorbance of GSH was measured at 510 nm to serve as the positive control. The percentage clearance effect was quantified as follows:

$$\begin{array}{c} \text{Hydroxyl radical scavenging activity}\;\left(\%\right)=\left(1-\frac{A_{\text{sample}}-A_{\text{control}}}{\;A_{\text{blank}}}\right)\\ \;\times 100\% \end{array}$$

where A_blank_ is water instead of sample; the A_sample_ is the absorbance of sample in the reaction; and the A_control_ is the absorbance of the salicylic acid.

### 2.6 LC–MS/MS identification of peptide sequences

Peptide identification of fractions with the highest antioxidant activity was conducted using liquid chromatography–tandem mass spectrometry (LC–MS/MS). The liquid chromatographic column (0.15 mm × 150 mm, RP‐C18, Column Technology Inc.) was equilibrated with a 95% solution of solution A (0.1% formic acid aqueous solution). Subsequently, the dissolved samples were loaded from the autosampler onto Zorbax 300SB‐C18 peptide traps (Agilent Technologies, Wilmington, DE, USA) and then subjected to gradient separation on the chromatographic column. The liquid‐phase gradient was programmed as follows: from 0 to 50 min, the linear gradient of liquid B (0.1% formic acid–acetonitrile aqueous solution) increased from 4% to 50%; from 50 to 54 min, the linear gradient of liquid B increased from 50% to 100%; and from 54 to 60 min, liquid B was held constant at 100%.

Sample fractions were separated using capillary high‐performance liquid chromatography (HPLC) and analyzed by mass spectrometry (MS) using a Q Exactive HF‐X Mass Spectrometer (Thermo Scientific) with an analysis time of 60 min, in positive ion detection mode. The mass‐to‐charge ratios of peptides and peptide fragments were collected as follows: 10 fragment profiles were acquired after each full scan (MS2 scan). Raw files of the mass spectrometry (MS) tests were retrieved from the respective databases using MaxQuant 1.5.5.1 software.

### 2.7 Network pharmacology

#### 2.7.1 Screening of potentially active ingredients

Peptide sequences identified via mass spectrometry (MS) were assessed for bioactivity using the Peptide Ranker server (https://distilldeep.ucd.ie/PeptideRanker/). Sequences with a score >0.5 underwent screening for toxicity (ToxinPred, https://webs.iiitd.edu.in/raghava/toxinpred/multi_submit.php), solubility (Innovagen, http://www.innovagen.com/proteomics‐tools), and isoelectric point (pI) prediction (https://web.expasy.org/protparam/).

#### 2.7.2 Drug–disease target screening

Peptide targets of action were acquired via the SwissTargetPrediction website (http://www.swisstargetprediction.ch). Targets associated with oxidative stress were gathered from the GeneCards database (https://www.genecards.org/). The intersection of antioxidant targets with peptide‐active targets was determined, and the overlapping targets were visualized using Wayne plots.

#### 2.7.3 PPI network construction and analysis

The overlapping targets identified in Section 2.7.2 were employed to construct the protein–protein interaction (PPI) network using the Search Tool for the Retrieval of Interacting Genes/Proteins (STRING) database (https://cn.string‐db.org/). Subsequently, the PPI network was visualized using Cytoscape 3.9.1 software. The algorithms of Betweenness (BC), Closeness (CC), and Degree (DC) in CentiScape 2.2 were applied to identify the central targets within the PPI network.

#### 2.7.4 GO and KEGG enrichment analysis

The central targets identified in Section 2.7.3 underwent Gene Ontology (GO) enrichment analysis and Kyoto Encyclopedia of Genes and Genomes (KEGG) pathway enrichment analysis using the Database for Annotation, Visualization, and Integrated Discovery (DAVID) database (https://david.ncifcrf.gov/summary.jsp).

#### 2.7.5 Molecular docking

The structures of the identified central targets were downloaded from the Research Collaboratory for Structural Bioinformatics Protein Data Bank (RCSB PDB) database (https://www.rcsb.org/). Peptide structures were generated using Chem3D 20.0 software. Molecular docking between the core targets associated with oxidative stress and peptides was conducted using AutoDock Vina software. Subsequently, the docking results were assessed based on the lowest binding energy and visualized using PyMOL software.

#### 2.7.6 Solid‐phase synthesis of peptides

The peptide sequences identified through screening underwent solid‐phase synthesis. Liquid chromatography (LC) and mass spectrometry (MS) were employed to assess their purity and molecular mass. Ultimately, peptide fractions with purities exceeding 95% and precise molecular masses were obtained.

### 2.8 Human hepatocellular carcinoma HepG2 cells assay

#### 2.8.1 Culture of human hepatocellular carcinoma HepG2 cells

Human hepatocellular carcinoma HepG2 cells were inoculated into Dulbecco's modified Eagle medium (DMEM) supplemented with 10% fetal bovine serum (FBS) and 1% double antibody. The cells were cultured in a carbon dioxide (CO_2_) incubator at 37°C and passaged every 2 days.

#### 2.8.2 Human hepatocellular carcinoma HepG2 cells cytotoxicity assay

The impact of the peptide on human hepatocellular carcinoma HepG2 cells viability was assessed utilizing the CCK‐8 assay. Human hepatocellular carcinoma HepG2 cells were seeded at a density of 5 × 10^4^ cells per well in 96‐well plates and incubated for 24 h. The medium was then removed, and peptide‐containing medium with varying concentrations (100–1000 μg/mL) was added for incubation for 24 h. Subsequently, the medium was aspirated and replaced with 100 μL of basal medium and 10 μL of CCK‐8 solution per well. Following a 2 h incubation period, the absorbance at 450 nm was measured using an enzyme‐linked immunosorbent assay (ELISA), and cell viability was determined by comparing the percentage of viable cells in the experimental group to that in the control group.

#### 2.8.3 Modeling of oxidative stress injury

Human hepatocellular carcinoma HepG2 cells in the logarithmic growth phase were harvested and inoculated at a density of 5 × 10^4^ cells per well in 96‐well plates, followed by 24 h of culture. The culture medium was subsequently removed. The injury was induced by treating the cells with a medium containing varying concentrations of H_2_O_2_, followed by the assessment of cell viability as described in Section 2.8.2.

#### 2.8.4 Protective effects of peptides against oxidative stress injury in human hepatocellular carcinoma HepG2 cells

Human hepatocellular carcinoma HepG2 cells in the logarithmic growth phase were seeded at a density of 5 × 10^4^ cells per well in 96‐well plates and cultured for 24 h. Following incubation, the culture medium was removed, and the cells were treated with peptide solutions of varying concentrations (200, 400, 600, 800, and 1000 μg/mL) prepared in a DMEM. Each well received 100 μL of the respective solution and was then incubated for an additional 24 h. Subsequently, the medium was replaced with fresh medium containing 800 μM H_2_O_2_, and the cells were exposed to this oxidative stressor for 3 h. The control group was treated with 100 μL of DMEM only. Cell viability was assessed using method 2.9.12.

### 2.9 Quantum chemical characterization of peptide structures

The software package GaussView 5.0 was utilized for the construction of the structural model of the synthetic peptide molecule. Frequency analysis and structural optimization were performed using Gaussian 09 software employing the density‐functional theory (DFT) B3LYP/6‐31G(d) basis set until achieving nonnegative vibrational frequencies and zero imaginary frequencies. The energy parameters (including total molecular energy, distribution of molecular frontier orbitals, and energy levels) and structural parameters (such as atomic Mulliken charge distribution and bond lengths) of the peptide molecules were determined through calculation.

### 2.10 Statistical analysis

Each experiment in this study was conducted in triplicate, and subsequent statistical analysis and graphical representation were conducted using the software packages SPSS 25.0 and Origin 9.0 Pro. The results were presented as the mean ± standard deviation (SD). Analysis of variance (ANOVA) was conducted, followed by comparisons of means using Duncan's multiple range test. Significance was determined at *p* < .05.

## 3 RESULTS AND DISCUSSION

### 3.1 Antioxidant activity of ultrafiltration fractions

**FIGURE 1:**
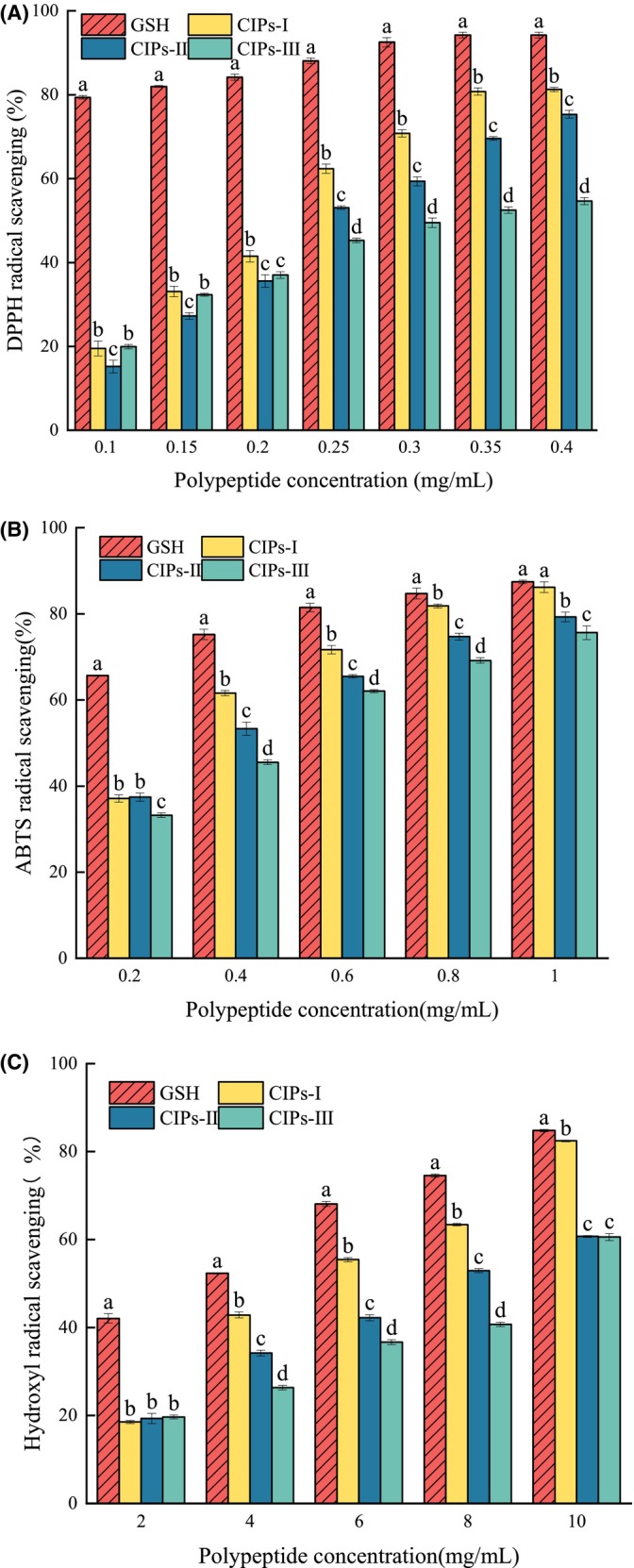
(A) DPPH radical scavenging activity of ultrafiltration fractions with different concentrations (CIPs‐I, MW < 3 kDa; CIPs‐II, MW 3–10 kDa; and CIPs‐III, MW > 3 kDa); (B) ABTS radical scavenging activity of ultrafiltration fractions at different concentrations; (C) Hydroxyl radical scavenging activity of ultrafiltration fractions at different concentrations. GSH was used as a positive control. Different letters indicate significant differences (*p* < .05).

Free radical‐mediated oxidative stress has been intricately linked to the etiology and pathogenesis of various human diseases, emerging as a significant contributor to conditions such as cardiovascular diseases and cancers, thereby exerting detrimental effects on human health (Chaudhary et al., 2023). The DPPH radical scavenging rate of each component showed a concentration‐dependent increase (Figure 1A). At equivalent concentrations, the CIPs‐I fraction demonstrated superior DPPH radical scavenging activity compared to the CIPs‐II and CIPs‐III fractions within the concentration range of 0.2–0.4 mg/mL (*p* < .05). The molecular mass of peptides correlates closely with their antioxidant activity. Peptides with lower molecular weights typically contain more hydrophobic amino acids. In the context of the DPPH radical scavenging reaction, which involves single‐electron transfer, these hydrophobic amino acids readily donate electrons to free radicals, resulting in heightened antioxidant capacity (Udenigwe et al., 2009). This phenomenon is evident in various plant‐derived antioxidant peptides. For instance, walnut protein antioxidant peptide (WPPH) was fractionated using a 3 kDa ultrafiltration membrane, with the fraction below 3 kDa demonstrating superior DPPH radical scavenging activity (60.25% at 2 mg/mL) (Chen et al., 2012). Similarly, during the isolation and purification of soybean dregs peptides, UAFP4, a peptide with a molecular weight of less than 1 kDa, exhibited the highest antioxidant capacity. The robust antioxidant activity of UAFP4 can be attributed to its abundance of hydrophobic amino acids, such as Pro, His, and Tyr, which are prevalent in its small peptide structure (Xie et al., 2024).

The half‐maximal inhibitory concentration (IC_50_) value of the CIPs‐I fraction was 0.204 ± 0.002 mg/mL, significantly lower than those of the CIPs‐II and CIPs‐III fractions (*p* < .05). Moreover, it demonstrated superior efficacy compared to previously reported peptides, such as those derived from sesame protein hydrolysis using a dual‐enzyme system, which exhibited an IC_50_ of 5.689 ± 0.078 mg/mL (Lu et al., 2019). The dual‐enzyme approach facilitated more comprehensive hydrolysis of sesame protein compared to single‐enzyme methods, resulting in the production of lower‐molecular‐weight peptides. These peptides not only displayed enhanced biological activity but also exhibited superior cell membrane permeability, enabling rapid absorption into the bloodstream for efficient uptake in the human body (Sun & Udenigwe, 2020).

The ABTS radical scavenging activity of each ultrafiltration fraction was also significant, primarily assessed by the ability of antioxidant peptides to engage in hydrogen atom transfer. The ABTS radical scavenging activity of the ultrafiltration fractions increased in a concentration‐dependent manner (Figure 1B), with the scavenging rate of the CIPs‐I fraction significantly higher than those of the other fractions (*p* < .05). The ABTS radical scavenging activity increased with the decrease in the molecular weight of the peptides and correlated with their composition, structure, and hydrophobicity, particularly those containing histidine (His) amino acids, which are associated with imidazole's antioxidant activity, hydrogen donor properties, lipid peroxyl radical trapping ability, and metal ion chelating ability (Sarmadi & Ismail, 2010). The IC_50_ of the CIPs‐I fraction was 0.377 ± 0.006%, significantly lower than those of the other fractions (*p* < .05).

Hydroxyl radicals, among the most reactive radicals, can form via the reaction between superoxide anions and hydrogen peroxide under metal ion conditions. Possessing high reactivity, hydroxyl radicals can engage in double‐bond addition reactions with aromatic compounds, generating hydroxycyclohexanediene radicals. The hydroxyl radical scavenging capacity of each ultrafiltration component exhibited a concentration‐dependent increase (Figure 1C). Within the concentration range of 4–10 mg/mL, the scavenging rate of the CIPs‐I fraction significantly surpassed those of other groups (*p* < .05). The presence of hydrogen atom donor compounds in the peptide likely contributes to the scavenging of hydroxyl radicals. The notable hydroxyl radical scavenging activity of the CIPs‐I fraction aids in mitigating free radical production in the human system, thereby contributing to human health protection. The IC_50_ of the CIPs‐I fraction was 4.953 ± 0.019%, markedly lower than those of the other fractions (*p* < .05). Other peptides retained through 3 KDa ultrafiltration membranes also demonstrated commendable hydroxyl radical scavenging abilities, such as the *Quasipaa spinosa* skin peptide of <3 KDa, with an IC_50_ value of 3.0 ± 0.3 mg/mL, exhibiting superior hydroxyl radical scavenging activity compared to that of the CIPs‐I fraction.

### 3.2 Secondary structure of ultrafiltration components

**FIGURE 2:**
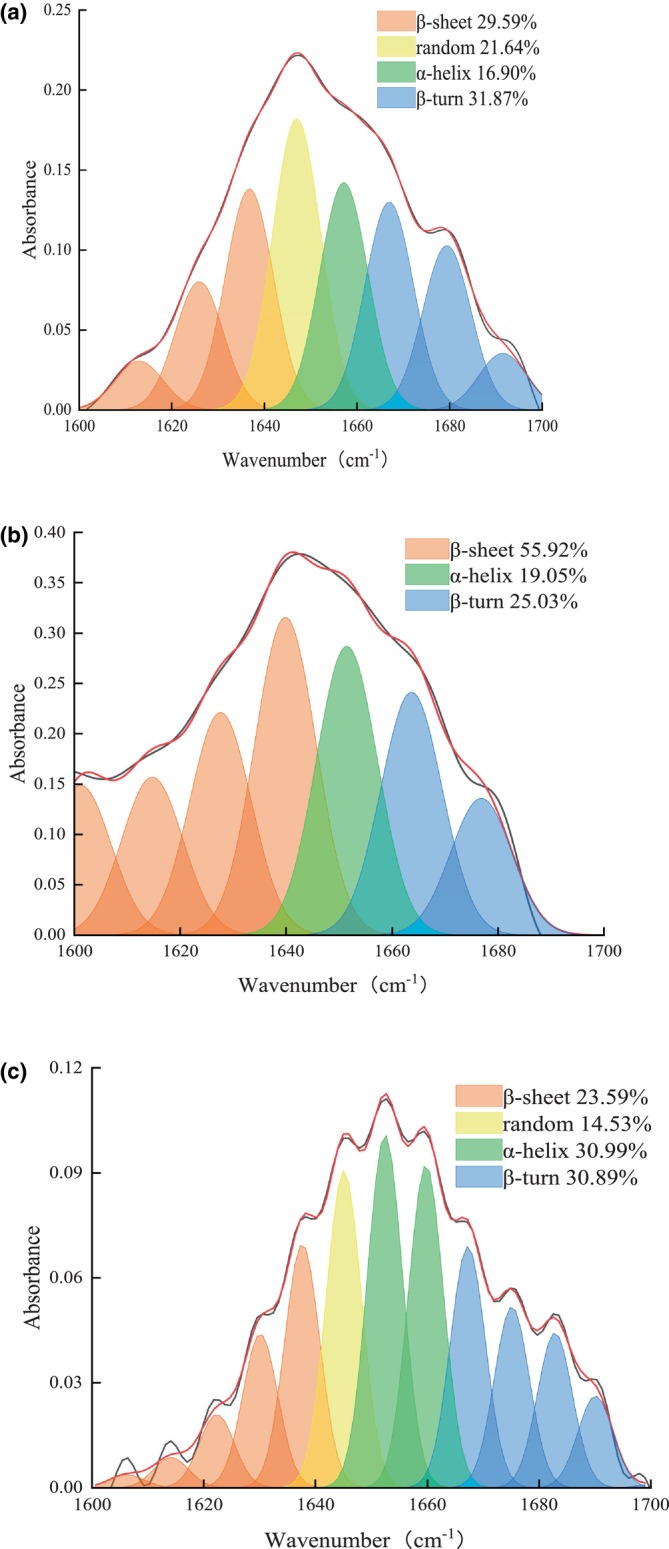
(a) Deconvolved and curve fitted bands of the FTIR spectra of amide I of CIPs‐I; (b) Deconvolved and curve fitted bands of the FTIR spectra of amide I of CIPs‐ II; (c) Deconvolved and curve fitted bands of the FTIR spectra of amide I of CIPs‐III.

The deconvolution curves were fitted to the amide I band of the ultrafiltration fraction using PeakFit software. Results revealed that the α‐helix proportion in the CIPs‐I fraction (Figure 2a) was 16.9%, lower than those in the CIPs‐II fraction (Figure 2b) (19.05%) and the CIPs‐III fraction (Figure 2c) (30.99%). During the protease‐catalyzed enzymatic hydrolysis of *I. polycarpa* Maxim. cake meal proteins, α‐helices and β‐helices underwent primary hydrolysis, causing gradual unfolding of the protein conformation and the emergence of numerous irregular structures. This unfolding process transitions the protein's ordered structure to a disordered state, exposing more hydrophobic groups and enhancing its antioxidant capacity. Ultrafiltration then separated and purified the proteolytic digest, leading to the migration of amino acids and hydrophobic groups toward fractions with smaller molecular weights, thereby increasing the disordered structure. Notably, the CIPs‐I fraction exhibited a maximum of 21.64% irregular curls, significantly higher than other fractions. Peptides with potent antioxidant activity typically possessed molecular weights ranging from 500 to 1800 Da and comprised five to nine amino acid residues. These peptides showcased structural characteristics conducive to antioxidant activity, featuring rich secondary structures. Hence, it's plausible to infer that CIPs‐I fractions, with molecular weights below 3 KDa, harbor an abundance of highly antioxidative short peptides and hydrophobic groups, endowing them with robust antioxidant properties.

### 3.3 Sephadex G‐25 column for separation of CIPs‐I fractions

**FIGURE 3:**
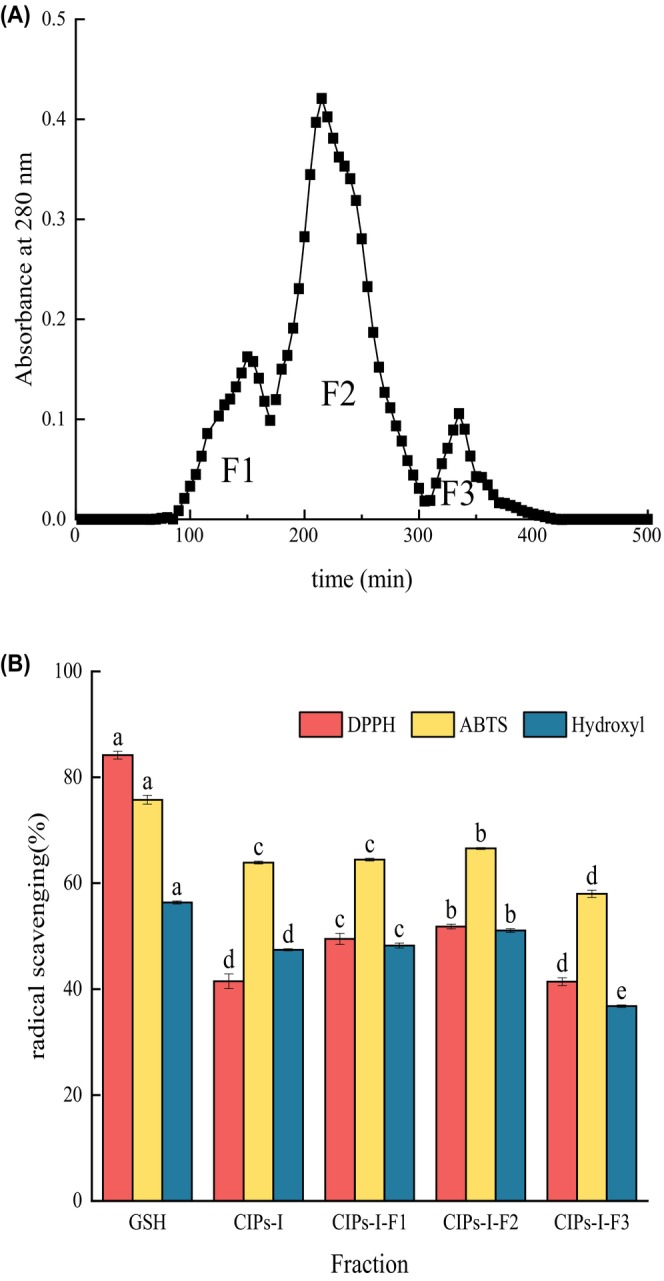
Size‐exclusion chromatogram and antioxidant activity of *Idesia polycarpa* Maxim. cake meal peptides. (a) Chromatograms of *I. polycarpa* Maxim. cake meal hydrolysate fraction with molecular weight <3 kDa (CIPs‐I) by Sephadex G‐25 gel filtration; (b) DPPH, ABTS, and hydroxyl radical scavenging activities of *I. polycarpa* Maxim. cake meal peptide fractions from G‐25 gel filtration.

The CIPs‐I fraction underwent successful separation into three subfractions via a Sephadex G‐25 column (Figure 3A). Upon evaluating their DPPH, ABTS, and hydroxyl radical scavenging capacities, it was evident that the antioxidant activities of the CIPs‐I‐F2 fractions surpassed those of the other subfractions (Figure 3B). Specifically, at a concentration of 0.2 mg/mL, the DPPH radical scavenging rate of the CIPs‐I‐F2 fraction reached 51.8 ± 0.42%, significantly exceeding those of the F1 fraction (49.48 ± 41.05%) and F3 fraction (41.4 ± 0.74%) (*p* < .05). Similarly, at concentrations of 0.5 and 5 mg/mL, the CIPs‐I‐F2 fraction exhibited superior performance in scavenging ABTS and hydroxyl radicals compared to the other fractions, with scavenging rates of 66.56 ± 0.19% for ABTS radicals and 51.06 ± 0.35% for hydroxyl radicals, both significantly higher than those of the other two fractions (*p* < .05). Thus, the CIPs‐I‐F2 fractions emerged as the most effective fraction for free radical scavenging among the peptides derived from *I. polycarpa* Maxim. cake meal.

### 3.4 Characterization of CIPs‐I‐F2 fractions by LC–MS/MS

**FIGURE 4:**
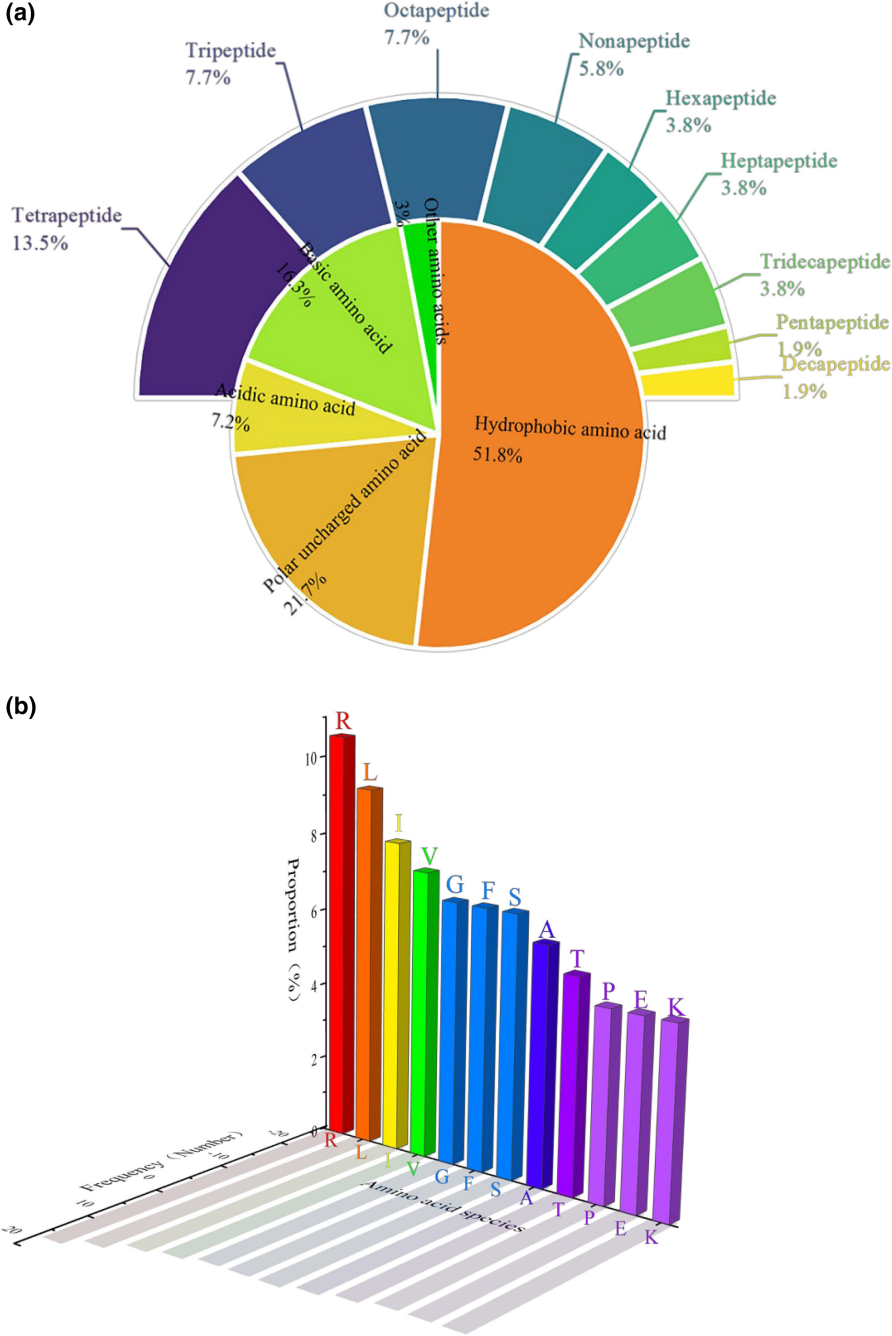
(a) Amino acid properties and distribution of peptides in CIPs‐I‐F2 fractions; (b) Amino acid occupancy and frequency of peptides in CIPs‐I‐F2 fractions.

Peptidomics, as a subfield within the realm of proteomics, is dedicated to investigating the composition, interactions, and properties of peptides found in extracts, while also delving into the origins and dynamics of the released peptides (Carrasco‐Castilla et al., 2012). The fractions of CIPs‐I‐F2 were identified using the LC–MS/MS technique, resulting in the acquisition of a total of 26 peptides (Table 1). These peptides predominantly ranged between 3 and 13 amino acid residues in length, exhibiting molecular weights spanning from 290.12 to 1451.79 Da. They were primarily comprised of tripeptides, tetrapeptides, and octapeptides, with tripeptides notably constituting the largest proportion at 13.5% (Figure 4a). Short peptide sequences exhibit superior antioxidant activity compared to longer peptides due to their tendency to possess a more stable structure and undergo easier degradation. These characteristics render them more efficacious antioxidants within organisms. Furthermore, the arrangement of amino acid residues within short peptide sequences also significantly influences their antioxidant activity (Nimalaratne et al., 2015). Within the CIPs‐I‐F2 fractions, peptides exhibit a predominant composition of hydrophobic amino acids, comprising 51.8% of the total, followed by polar uncharged amino acids at 21.7%. The robust interactions facilitated by highly hydrophobic amino acids within the peptide chain enable the formation of stable hydrogen bonds and salt bridges with other amino acid residues. Consequently, these peptides possess an enhanced ability to engage with free radicals, thereby neutralizing them and safeguarding biological macromolecules from damage. The prevalence of highly hydrophobic amino acid residues characterizes antioxidant peptides, exemplifying their distinctive feature (Ahmed et al., 2015). Analogously, a substantial presence of highly hydrophobic amino acids characterizes other plant‐derived antioxidant peptides. In a study investigating potato antioxidant peptides prepared through three distinct methods—nonultrasonicated conventional enzymatic hydrolysis (C), energy‐divergent ultrasound (EDU), and gathered ultrasound (EGU)—it was observed that the proportions of hydrophobic peptides were remarkably high, constituting 89.66%, 91.43%, and 98.11% of the total peptides, respectively (Liu et al., 2023). These findings underscore the dominance of hydrophobic peptides within these samples and highlight the efficacy of ultrasound assistance in augmenting the proportions of hydrophobic peptides. Thus, the enrichment of highly hydrophobic peptides and amino acid residues emerges as a consistent hallmark of antioxidant peptides. The strategic localization of hydrophobic amino acids at the N‐terminal and C‐terminal regions of the peptide chain plays a pivotal role in conferring antioxidant activity, particularly in peptides devoid of internal hydrophobic residues (Habinshuti et al., 2020). Analysis of maize‐derived antioxidant peptides revealed a substantial predominance of hydrophobic amino acids, constituting 66% of the total, with widespread distribution at both ends of the peptide chain, collectively representing approximately 65%. Notably, the amino acid L exhibited the highest frequency, occurring 93 times (20.90%) at the N‐terminus and 64 times (14.35%) at the C‐terminus, followed by P and F.

**TABLE 1:** Peptide sequences and basic information in CIPs‐I‐F2 fractions.

Number	Sequence	Length	Mass
1	LIYR	4	563.34313
2	LLVR	4	499.34822
3	LVVVLR	6	697.48505
4	VVLR	4	485.33257
5	ITFQ	4	507.2693
6	LYS	3	381.18999
7	EDR	3	418.18121
8	GPR	3	328.1859
9	NSA	3	290.12263
10	VRAP	4	441.26997
11	FREY	4	613.28601
12	LAFR	4	505.30127
13	ILEAHK	6	709.41227
14	KVFER	5	677.38606
15	LITFGAA	7	691.39048
16	LSGGSCR	7	678.31191
17	INKISWKI	8	1000.607
18	LGHPWGNAPG	10	1004.4828
19	ISKPTWADF	9	1063.5338
20	GWGTTPLM	8	861.40547
21	NIIRDKKRC	9	1144.6499
22	YGRERCNN	8	1010.4352
23	SVEFQIVSF	9	1054.5335
24	AFTFLVRD	8	967.51272
25	TEGTVRKMTYPFI	13	1541.7912
26	NQSLSEAWSKIPE	13	1487.7256

The composition of component amino acids in CIPs‐I‐F2 encompasses a diverse array of 20 distinct amino acids. Notably, amino acids R, L, I, V, G, F, and S emerge with considerable frequency, constituting 10.56%, 9.32%, 8.07%, 7.45%, and 6.83%, respectively (Figure 4b). Importantly, the predominant presence of hydrophobic amino acids characterizes this composition. It has been documented that hydrophobic amino acids can synergistically enhance antioxidant effects when combined with aromatic amino acids. For instance, the incorporation of amino acids like L and P at the N‐terminus of H–H substantially bolsters the antioxidant prowess of peptides (Wen et al., 2020). Furthermore, aromatic amino acids, such as F, Y, and H, possess the ability to neutralize free radicals by donating electrons, thereby engendering potent antioxidant activity (Najafian & Babji, 2015). It is notable that R, classified as a basic amino acid, exhibits the highest prevalence within the CIPs‐I‐F2 fractions, occurring 17 times, yet no antioxidant activity has been attributed to R and S thus far. Structurally, the R side chain features multiple nitrogen atoms, capable of acting as proton donors or acceptors, while the S side chain contains hydroxyl groups, both properties suggesting the potential for antioxidant activity (Zhou et al., 2024). Given recent findings demonstrating significant anti‐inflammatory effects of R and S, the next research frontier should prioritize investigating their role in antioxidation.

### 3.5 Screening of potentially active peptides in CIPs‐I‐F2 fractions

**FIGURE 5:**
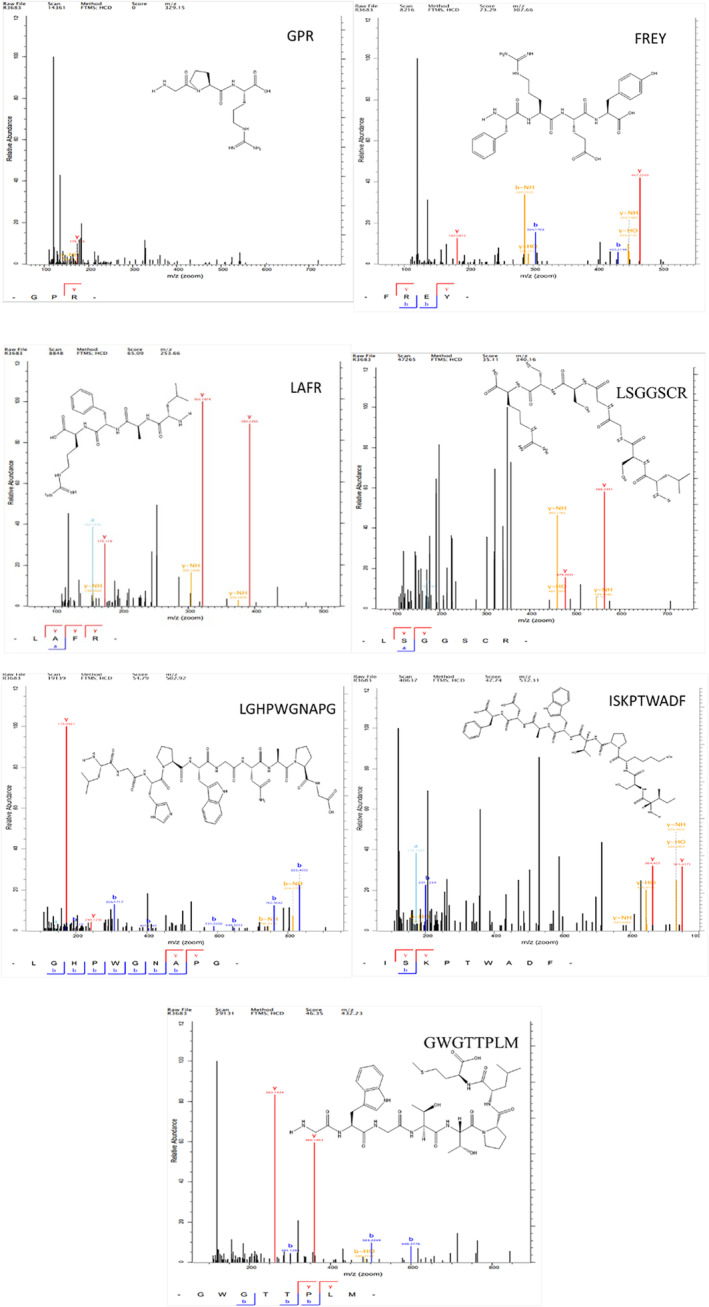
Two‐dimensional (2D) structure and mass spectrometry of active peptides from *Idesia polycarpa* Maxim. cake meal.

The bioinformatics characterization of CIPs‐I‐F2 fractions involved assessing the bioactivity of its component peptides using the Peptide Ranker server. A Peptide Ranker score closer to 1 indicates higher bioactivity (ranging from 0 to 1). For this study, a threshold value of 0.5 was set, with peptides scoring >0.5 considered potentially biologically active (Zheng et al., 2023). Seven peptides were identified from the CIPs‐I‐F2 fraction, not documented in the Bioactive Peptides (BIOPEP) database, and thus classified as novel bioactive peptides. Predicting the toxicity, solubility, and isoelectric point of these peptides was crucial for determining their physicochemical properties and safety (Table 2). Results indicated good water solubility for all peptides except LGHPWGNAPG and GWGTTPLM, with all seven peptides demonstrating nontoxic characteristics. Visualizations of the two‐dimensional (2D) structures and secondary mass spectra were generated for the seven active peptides (Figure 5).

**TABLE 2:** Basic information on potentially active peptides in *Idesia polycarpa* Maxim. cake meal.

Number	Sequence	Solubility	Isoelectric point	Poisonous	Stability index
A	GPR	Better	10.84	No	–
B	FREY	Better	6.6	No	–
C	LAFR	Better	10.84	No	–
D	LSGGSCR	Better	8.86	No	72.77
E	LGHPWGNAPG	Mediocre	7.81	No	3.64
F	ISKPTWADF	Better	6.64	No	−50.70
G	GWGTTPLM	Mediocre	3.71	No	0.60

### 3.6 Network pharmacology analysis

#### 3.6.1 Results of target screening and PPI network analysis

**FIGURE 6:**
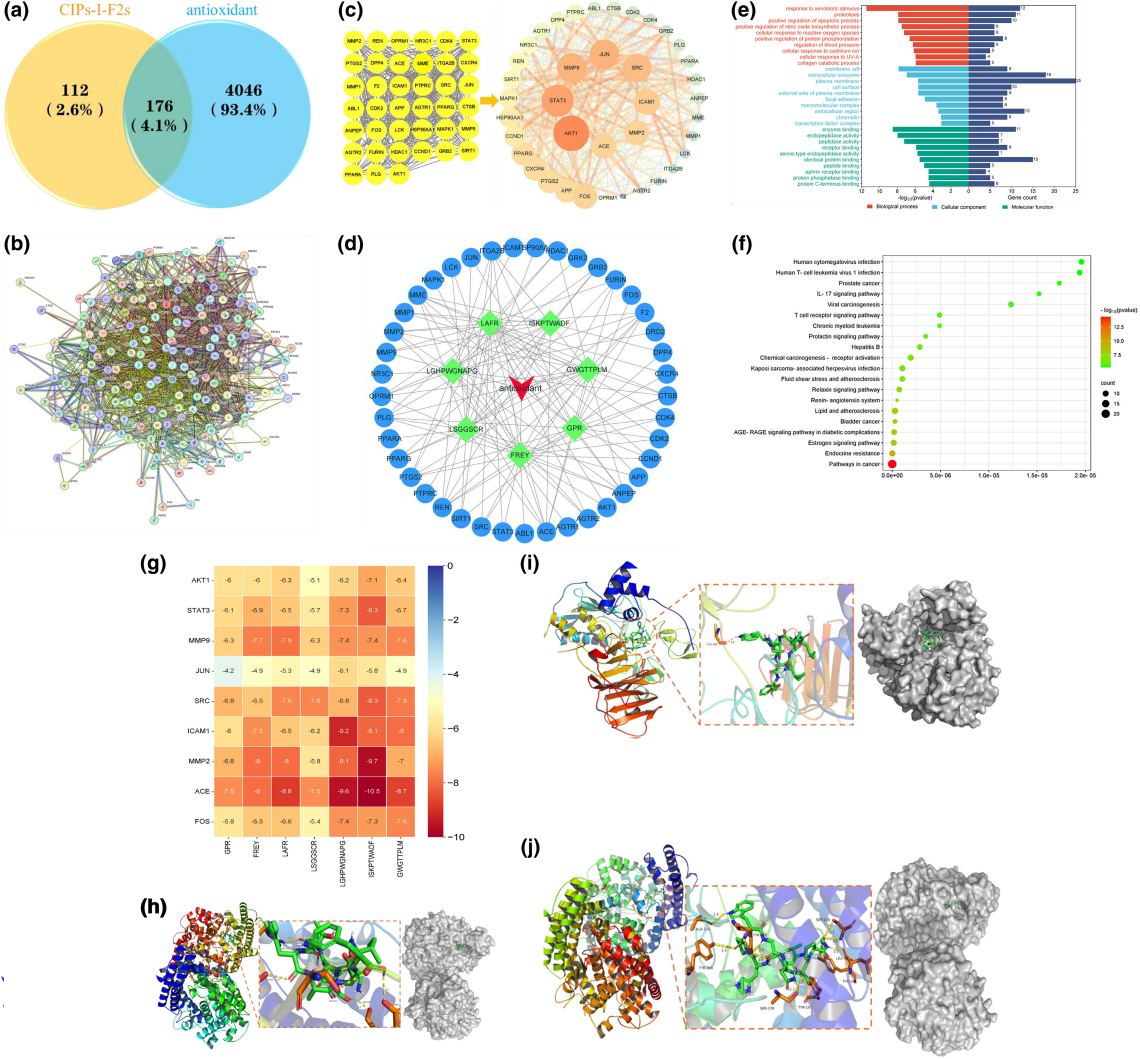
(a) Venn diagram of the intersection of potentially active targets of CIPs‐I‐F2s with antioxidant targets; (b) Intersection gene PPI map of “CIPs‐I‐F2s‐antioxidant potential target”; (c) “CIPs‐III‐F2s‐antioxidant Core Targets” and visualization analysis; (d) Antioxidant‐peptides‐pathway; (e) Functional analysis of CIPs‐I‐F2s interfering with antioxidant key targets GO (top 10); (f) Enrichment analysis of KEGG metabolic pathway, a key target of antioxidant, intervened by CIPs‐I‐F2s (top 20); (g) Heat map of free binding energy of CIPs‐I‐F2s docking with core target molecules; (h) Interaction between ISKPTWADF and ACE active site molecules; (i) Interaction between ISKPTWADF and MMP2 active site molecules; (j) Interaction between LGHPWGNAPG and ACE active site molecules.

The SwissTargetPrediction database was utilized to mine seven active peptides (CIPs‐I‐F2s), resulting in 112 unique targets after eliminating duplicates. A search for oxidative stress targets was conducted in the GeneCards database, yielding a total of 4046 targets with “antioxidant” as the keyword. Intersection analysis of the targets of CIPs‐I‐F2s and antioxidant targets was performed using a Venn diagram, revealing 122 intersecting targets, with a target mapping rate of 4.1% (Figure 6a). These targets are considered potential targets of CIPs‐I‐F2s for the treatment of oxidative stress. The 176 potential antioxidant targets were imported into the STRING database, generating a protein interaction network graph depicting the relationship between CIPs‐I‐F2s and antioxidants (Figure 6b). The network comprises 175 nodes and 1993 edges, indicating a dense network with numerous connections. With a relatively high average node degree of 22.8 and an average local clustering coefficient of 0.533, the network suggests frequent interactions among nodes and the formation of stable clusters.

#### 3.6.2 Core target screening

Further analysis of the PPI network was conducted by analyzing the intersecting targets using the STRING database and Cytoscape 3.9.1 topology data. A total of 39 core targets were identified (Table 3). After screening, the network comprised 39 nodes and 416 edges. The importance of the 39 core target points was assessed based on Degree value and combined score value (Figure 6c). Nodes with larger Degree values were represented by larger areas and darker colors, while nodes with larger combined score values were connected by thicker lines. The top nine targets in the PPI network were screened using the BC, CC, and DC algorithms for the following proteins: AKT1 (Degree: 103), STAT3 (Degree: 77), MMP9 (Degree: 80), JUN (Degree: 74), SRC (Degree: 89), Intercellular Adhesion Molecule 1 (ICAM1) (Degree: 59), Matrix metalloproteinase 2 (MMP2) (Degree: 56), angiotensin‐converting enzyme (ACE) (Degree: 46), and transcription factor AP1 (FOS) (Degree: 60).

**TABLE 3:** CIPs‐I‐F2s interferes with antioxidant core targets.

Number	Uniprot	Genetics	Protein name
1	P00519	ABL1	Tyrosine‐protein kinase ABL1
2	P28482	MAPK1	MAP kinase ERK2
3	P24385	CCND1	Cyclin‐dependent kinase 4/cyclin D1
4	P11802	CDK4	Cyclin‐dependent kinase 4/cyclin D1
5	P40763	STAT3	Signal transducer and activator of transcription 3
6	P24941	CDK2	Cyclin‐dependent kinase 2/cyclin A
7	P05067	APP	Beta amyloid A4 protein
8	P15144	ANPEP	Aminopeptidase N
9	P01100	FOS	Transcription factor AP1
10	P07900	HSP90AA1	Heat shock protein HSP 90‐alpha
11	P05412	JUN	Transcription factor AP1
12	P31749	AKT1	Serine/threonine‐protein kinase AKT
13	Q13547	HDAC1	Histone deacetylase 1
14	P61073	CXCR4	C‐X‐C chemokine receptor type 4
15	P06239	LCK	Tyrosine‐protein kinase LCK
16	P08575	PTPRC	Leukocyte common antigen
17	P08473	MME	Neprilysin
18	P12931	SRC	Tyrosine‐protein kinase SRC
19	P62993	GRB2	Growth factor receptor‐bound protein 2
20	P12821	ACE	Angiotensin‐converting enzyme
21	Q96EB6	SIRT1	NAD‐dependent deacetylase sirtuin 1
22	P08253	MMP2	Matrix metalloproteinase 2
23	P04150	NR3C1	Glucocorticoid receptor
24	P08514	ITGA2B	Integrin alpha‐IIb/beta‐3
25	P05362	ICAM1	Intercellular adhesion molecule (ICAM‐1), Integrin alpha‐L/beta‐2
26	P00797	REN	Renin
27	P37231	PPARG	Peroxisome proliferator‐activated receptor gamma
28	P07858	CTSB	Cathepsin (B and K)
29	Q07869	PPARA	Peroxisome proliferator‐activated receptor alpha
30	P35354	PTGS2	Cyclooxygenase‐2
31	P03956	MMP1	Matrix metalloproteinase 1
32	P00747	PLG	Plasminogen
33	P00734	F2	Thrombin
34	P14780	MMP9	Matrix metalloproteinase 9
35	P09958	FURIN	Furin
36	P27487	DPP4	Dipeptidyl peptidase IV
37	P50052	AGTR2	Angiotensin II receptor
38	P30556	AGTR1	Type‐1 angiotensin II receptor
39	P35372	OPRM1	Mu opioid receptor (by homology)

Serine/threonine‐protein kinase AKT (AKT1), as a crucial signaling molecule, plays a pivotal role in regulating cell growth, division, and apoptosis. Its activation is primarily induced by insulin and a diverse array of growth and survival factors. Studies have demonstrated that the E17K mutation in the AKT1 gene profoundly influences the regulation of metabolic, inflammatory, and redox genes in meningiomas. The AKT1–E17K mutation in meningiomas promotes the expression of hexokinase 2 (HK2) while mitigating oxidative stress. The AKT1–E17K mutation can contribute to decreasing intracellular ROS accumulation, concomitant with elevated levels of Nrf2 and the antioxidant enzyme superoxide dismutase 1 (SOD1) (Singh et al., 2024). STAT3, belonging to the Signal transducer and activator of transcription (STAT) family of proteins, undergoes phosphorylation activation in response to various cytokines and growth factors. Overexpression of STAT3 leads to a reduction in ROS production, suppression of mitochondria‐mediated apoptosis, and preservation of the closed state of the mitochondrial permeability transition pore (Boengler et al., 2010). MMP9 and MMP2 are members of the matrix metalloproteinases (MMPs) protease family. Upon MMP release, particularly MMP9, heightened activity accelerates the degradation of extracellular matrix (ECM) components and stimulates the release of inflammatory cytokines and chemokines. This inflammatory response serves as a signal directing immune cells to the site of damage, thereby exacerbating tissue damage and promoting the generation of reactive oxygen species. SRC kinase, the most extensively researched member of the SRC family, has emerged as a focal point in biological and medical investigations, owing to its pivotal role in cellular signaling and associated pathologies. SRC can bind to activated focal adhesion kinase (FAK), forming the FAK–Src complex, thereby facilitating the activation of signaling pathways. The researchers discovered that cyclosporine A (CsA) shields human trophoblast‐like JEG‐3 cells from H_2_O_2_‐induced oxidative damage by triggering the FAK/Src signaling pathway. Additionally, they observed that this pathway activation bolsters the antioxidant capacity of the cells, consequently mitigating oxidative stress‐induced damage (Tang et al., 2019).

#### 3.6.3 “Antioxidant‐peptides‐pathway” network diagrams

The “Antioxidant‐peptides‐pathway” network was constructed using Cytoscape 3.9.1 (Figure 6d). The network comprises 49 nodes and 133 edges, with an average Degree value of 5.429. The peptides containing F, L, and R exhibit notably high Degree values, with an average Degree value of 5.429. Seven peptides possess Degree values exceeding 5.429, suggesting their high complementarity. These peptides can target multiple antioxidant core targets, thus engendering synergistic effects across various pathways, thereby enhancing therapeutic efficacy. These peptides can concurrently address oxidative stress, inflammatory responses, apoptosis, and other pathological aspects, mitigating disease progression from diverse angles. Moreover, they exhibit synergistic effects in targeting the antioxidant process, augmenting each other's antioxidant effects.

#### 3.6.4 GO functional enrichment analysis and KEGG pathway analysis

To comprehensively understand the roles of the seven active peptide therapeutic targets in gene function and signaling pathways, we will analyze the intersecting targets through the GO function (Figure 6e) and KEGG (Figure 6f) pathway analysis using the DAVID database. The GO pathway comprises a total of 398 entries, with 280 entries categorized under Biological Process (BP), predominantly enriched for functions, such as response to xenobiotic stimulus, proteolysis, positive regulation of apoptotic process, etc. The Cell Composition (CC) section includes 46 entries, primarily enriched for membrane raft, extracellular exosome, plasma membrane, etc., while the Molecular Function (MF) section consists of 72 entries, mainly enriched for enzyme binding, endopeptidase activity, peptidase activity, etc. GO function enrichment analysis aids in understanding the regulatory roles of peptide therapeutic targets in organisms and their association with disease occurrence. Concurrently, the analysis of molecular functions, such as enzyme activity, ion channels, receptors, etc., aids in comprehending the mechanisms of action of peptide therapeutic targets at the molecular level and provides a crucial foundation for drug design.

The KEGG pathway encompasses a total of 120 entries, predominantly focusing on cancer pathways, such as the mitogen‐activated protein kinase (MAPK) pathway, phosphoinositide‐3‐kinase/protein kinase B/mammalian target of rapamycin (PI3K/Akt/mTOR) axis, STAT3 pathway, nuclear factor kappa B (NF‐κB) pathway, and more. Among these, the c‐MET (c‐mesenchymal–epithelial transition factor) pathway holds a distinctive position in oncology due to its capacity to activate numerous mitotic signaling cascades (Mohan et al., 2024). Inhibiting these pathways has emerged as a promising strategy for treating human cancer. Additionally, the KEGG pathway includes Endocrine resistance, where endocrine therapy can impede tumor growth by adjusting hormone levels or disrupting hormone signaling pathways. It also covers pathways, such as the estrogen signaling pathway, advanced glycation end products (AGE)–receptor for AGE (RAGE) signaling pathway in diabetic complications, Lipid and atherosclerosis, among others.

#### 3.6.5 Molecular docking

The core targets within the PPI network, including AKT1, STAT3, MMP9, JUN, SRC, ICAM1, MMP2, ACE, and FOS, were chosen for molecular docking with the CIPs‐I‐F2s (GPR, FREY, LAFR, LSGGSCR, LGHPWGNAPG, ISKPTWADF, and GWGTTPLM) using the AutoDock Vina software. The heat map visualized the lowest free binding energy for each group of docked molecules, with darker colors indicating stronger binding ability (Figure 6g). The antioxidant activity of each peptide was thoroughly assessed by calculating the total binding energy of the seven peptides for each target. The binding energies ranked in descending order were ISKPTWADF > LGHPWGNAPG > GWGTTPLM > LAFR > FREY > GPR > LSGGSCR. ISKPTWADF exhibited the highest total binding energy at −72.5 kcal/mol, while LSGGSCR demonstrated the lowest total binding energy at −54.5 kcal/mol.

ISKPTWADF demonstrates the strongest binding affinity to the ACE target, reaching −10.5 kcal/mol. The optimal docking pose involves forming five hydrogen bonds with the amino acids ARG‐381, TYR‐369, SER‐333, SER‐39, and ASP‐43 (Figure 6h). Subsequently, the binding energy of ISKPTWADF to the MMP2 target is −9.7 kcal/mol, with the optimal docking pose forming a hydrogen bond with the amino acid CYS‐390, with a bond length of 1.8 Å (Figure 6i). Finally, LGHPWGNAPG binds to the ACE target with a binding energy of −9.6 kcal/mol. It forms one hydrogen bond each with the amino acids ALA‐334, TYR‐369, SER‐378, TYR‐197, TYR‐186, and LEU‐98, two hydrogen bonds with the amino acid SER‐100, and three hydrogen bonds with the amino acid TYR‐338 (Figure 6j).

### 3.7 Verification of purity of synthetic peptides

**FIGURE 7:**
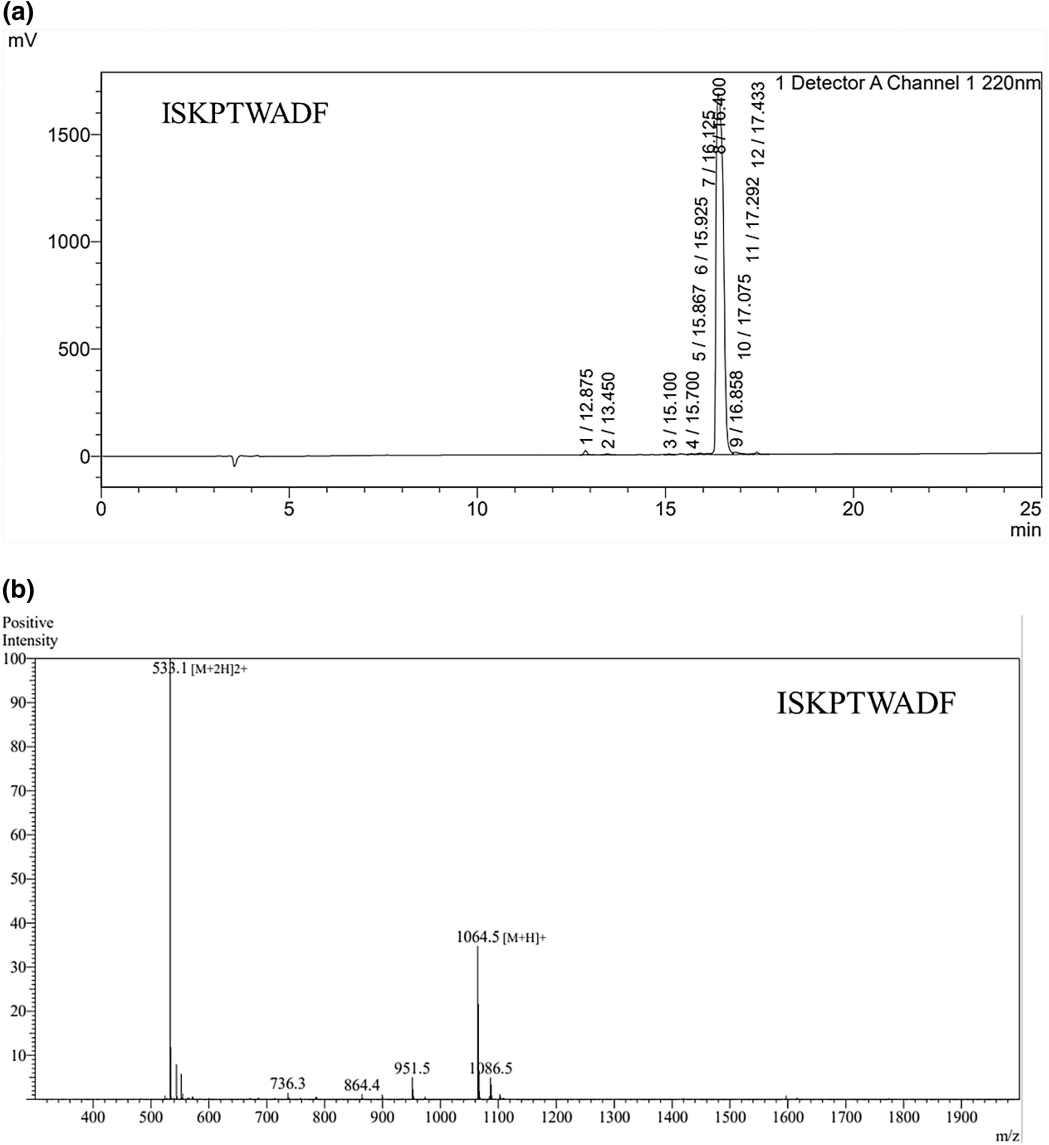
(a) The purity of identification by RP‐HPLC of peptide ISKPTWADF; (b) Molecular weight (MW) of ESI‐MS identification peptide ISKPTWADF.

The purity of ISKPTWADF was assessed using reversed phase‐high‐performance liquid chromatography (RP‐HPLC), where peaks in the chromatogram were identified by retention time. The HPLC purity of ISKPTWADF was determined based on the area of the target peptide, resulting in a purity of 97.589% (Figure 7a). Additionally, electrospray ionization mass spectrometry (ESI‐MS) was utilized to determine the mass‐to‐charge ratios (m/z) of individual ionized analytes. The observed molecular weight (MW) of ISKPTWADF was found to be 1064.2, which closely matched the theoretical MW (Figure 7b).

### 3.8 Protective effect of ISKPTWADF on oxidatively damaged human hepatocellular carcinoma HepG2 cells

#### 3.8.1 Modeling of oxidative damage in human hepatocellular carcinoma HepG2 cells

**FIGURE 8:**
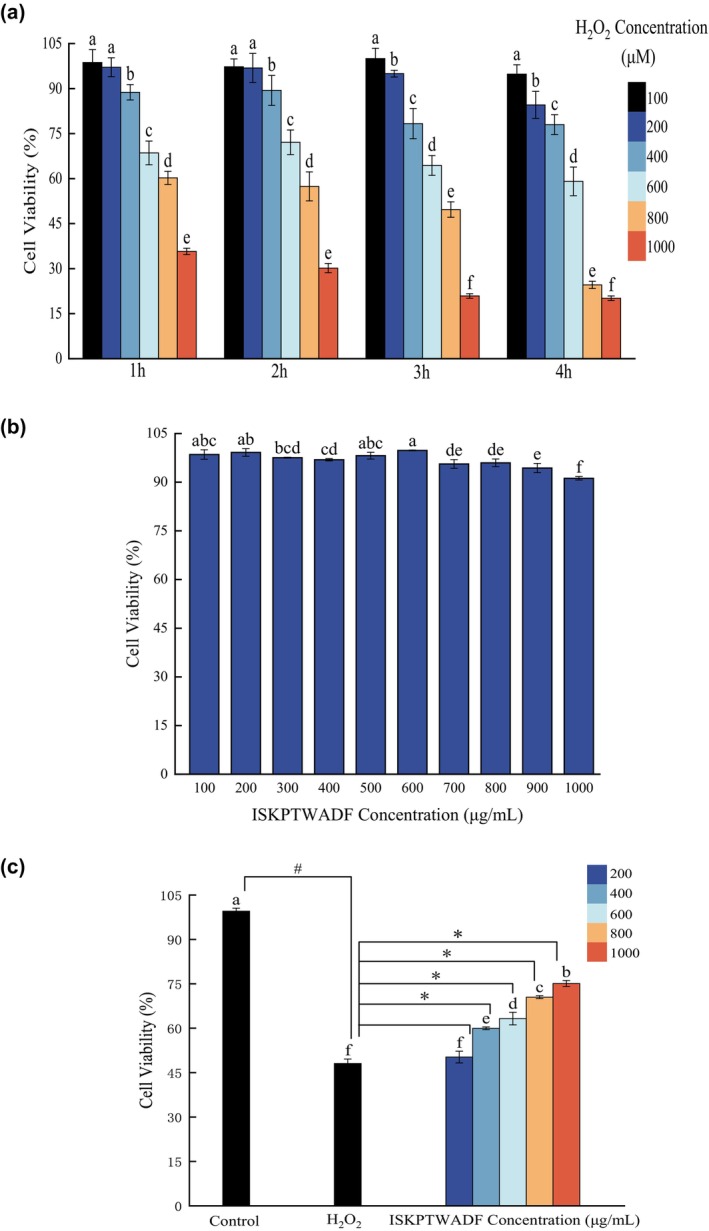
(a) Effect of H_2_O_2_ concentration and time on the survival of HepG2 cells; (b) Effect of ISKPTWADF on HepG2 cytotoxicity; (c) Protective effect of ISKPTWADF against H_2_O_2_‐induced oxidative damage in HepG2 cells. # indicates a significant difference between the H_2_O_2_ group and the control group; * indicates a significant difference between the H_2_O_2_ group and the sample group (p < .05).

Hydrogen peroxide (H_2_O_2_) exhibits high oxidizing properties, facilitating its permeation through the cell membrane and entry into the cellular interior. Consequently, H_2_O_2_ was selected as the inducer for establishing the oxidative damage model in human hepatocellular carcinoma HepG2 cells. The optimal conditions for inducing H_2_O_2_‐mediated cellular oxidative injury were ascertained by assessing cell viability under varying concentrations and durations of H_2_O_2_ exposure. The cellular median lethal dose (LD50) level was typically acknowledged as the optimal intervention criterion. Within the concentration range of 100–1000 μM, cell viability exhibited a dose‐dependent decline with increasing H_2_O_2_ concentration, whereas cellular activity progressively diminished with prolonged induction periods (Figure 8A). At a H_2_O_2_ concentration of 800 μM and an induction period of 3 h, the survival rate of human hepatocellular carcinoma HepG2 cells was 49.68 ± 2.57%, approximating the cellular LD50 threshold. Hence, this condition was selected for establishing the oxidative damage model in human hepatocellular carcinoma HepG2 cells.

#### 3.8.2 Protective effects of ISKPTWADF against human hepatocellular carcinoma HepG2 cells injury

In the concentration range of 100–1000 μg/mL, the cell viability exhibited minimal variation with increasing peptide concentration (Figure 8B), with a cell survival rate of 91.02 ± 0.56% at a peptide concentration of 1000 μg/mL. These findings suggest that ISKPTWADF does not influence human hepatocellular carcinoma HepG2 cells survival within the 100–1000 μg/mL concentration range. Different concentrations of ISKPTWADF demonstrated protective effects on human hepatocellular carcinoma HepG2 cells (Figure 8C). The cell survival rate in the H_2_O_2_ group was 48.09 ± 1.51%, indicating proximity to the cell LD50 condition and confirming successful model induction. Notably, in the experimental group, ISKPTWADF exhibited a dose‐dependent protective effect against H_2_O_2_‐induced oxidative damage in human hepatocellular carcinoma HepG2 cells. Within the concentration range of 400–1000 μg/mL, significant differences were observed between the experimental and H_2_O_2_ groups. At a concentration of 1000 μg/mL, the cell survival rate increased to 75.12 ± 0.99%, indicating the significant protective capability of ISKPTWADF against H_2_O_2_‐induced oxidative damage in human hepatocellular carcinoma HepG2 cells.

### 3.9 Quantum chemical analysis of the conformational relationships of ISKPTWADF

**FIGURE 9:**
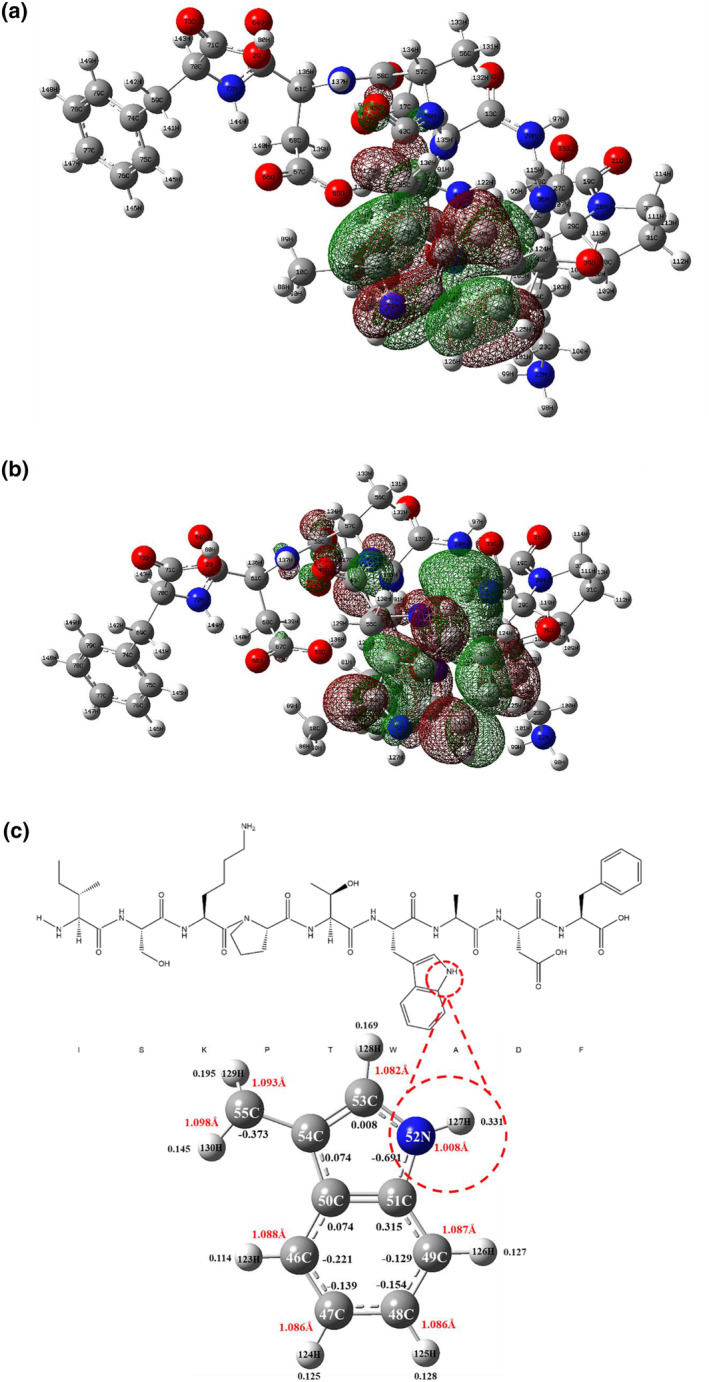
(a) HOMO distribution of ISKPTWADF; (b) LOMO distribution of ISKPTWADF; (c) Visualization of HOMO‐active sites of ISKPTWADF.

By the principles of frontier molecular orbital theory, the lowest unoccupied molecular orbital (LUMO) is positioned at a lower energy level and exhibits stronger electron‐binding tendencies, facilitating electron acceptance. In intermolecular interactions, the energy gap between the highest occupied molecular orbital (HOMO) and LUMO governs the character of the chemical reaction exhibited by the molecule. Indeed, when the energy of the HOMO is relatively high, the molecule tends to donate electrons readily, exhibiting oxidative behavior. Conversely, when the energy of the LUMO is low, the molecule is inclined to accept electrons easily, displaying reductive behavior. This relationship between molecular orbitals and energy parameters determines the conformational behavior of the molecule, which can be analyzed through careful examination of energy levels and molecular orbital structures (Igbokwe et al., 2024). The HOMO of ISKPTWADF predominantly resides on the indole ring of tryptophan, exhibiting a calculated value of −0.22334 Hartree (Figure 9a). The LOMO exhibits greater dispersion within the molecular structure, primarily encompassing the peptide bond, benzene ring, and indole ring of tryptophan, with a calculated value of −0.04188 Hartree (Figure 9b). Upon interaction with free radicals, the indole ring of tryptophan in ISKPTWADF is prone to electron loss. Studies have demonstrated that variations in charge within chemical bonds dictate site activity. Molecular bonding theory suggests that weaker attractions exist between elongated hydrogen atoms and negatively charged atoms, rendering the molecule susceptible to hydrogen atom loss and enhancing its antioxidant capacity (Lin et al., 2023). ISKPTWADF comprises 149 atoms, with 61 bearing negative charges, predominantly situated on carbon (C), nitrogen (N), and oxygen (O) atoms. Positive charges typically reside on H atoms. Mulliken charges are pivotal in intermolecular interactions, including hydrogen bonding, ionic bonding, and van der Waals forces. They act as hydrogen bond acceptors, exemplifying their significance.

Antioxidants primarily target free radicals within specific regions of the HOMO. Thus, it's crucial to analyze the charge distribution and bond lengths of Mulliken atoms within this orbital region. The analysis reveals that negative charges primarily accumulate on C and N atoms. Moreover, a significant difference in charge between adjacent atoms facilitates the transfer of extranuclear electrons, indicating molecular active sites. Within the HOMO of ISKPTWADF, the largest disparity in charge exists at 52N‐127H, with a bond length of 1.008 Å (Figure 9c). Consequently, it is inferred that the active site of ISKPTWADF resides at 52N‐127H within the tryptophan indole ring. Interestingly, among the seven antioxidant peptides derived from Corn gluten meal protein, the active sites are also situated within the tryptophan indole ring (Wang et al., 2024). Specifically, the active sites of AWF, FAW, LWQ, WIY, YLW, and LAYW may be positioned at 13N‐43H, 25N‐53H, 16N‐50H, 8N‐42H, 29N‐63H, 34N‐73H, and 23N‐59H on the tryptophan indole ring, respectively. These sites are presumed to act as hydrogen donors in scavenging free radicals, thus contributing to their antioxidant efficacy.

## 4 CONCLUSION

In this study, peptides were initially separated and purified through ultrafiltration of *I. polycarpa* Maxim. cake meal. The findings revealed that the CIPs‐I fraction exhibited the strongest antioxidant activity at <3 KDa. Subsequently, further purification on Sephadex G‐25 gel chromatographic columns demonstrated that the antioxidant activity of the CIPs‐I‐F2 fractions surpassed that of the other fractions. The identification of the CIPs‐I‐F2 fractions was conducted using LC–MS/MS. Seven of these peptides were screened to identify their main targets of antioxidant action using network pharmacology. These targets include AKT1, STAT3, MMP9, JUN, SRC, ICAM1, MMP2, ACE, and FOS. The principal pathways associated with their action involve cancer, endocrine regulation, and estrogen‐related pathways. The molecular docking results revealed that the peptide ISKPTWADF exhibited the highest total binding energy to the target, measuring at −72.5 kcal/mol. Subsequently, it was synthesized in vitro through solid‐phase synthesis. ISKPTWADF demonstrated a dose‐dependent protective effect against H_2_O_2_‐induced oxidative damage in human hepatocellular carcinoma HepG2 cells. At a peptide concentration of 1000 μg/mL, the cell survival rate reached 75.12 ± 0.99%, indicating significant protection of human hepatocellular carcinoma HepG2 cells from H_2_O_2_‐induced oxidative damage by ISKPTWADF. Quantum chemistry analysis revealed that the main active site of ISKPTWADF was located within the tryptophan indole ring at position 52N‐127H. These findings lay a certain foundation for the subsequent development of *I. polycarpa* Maxim. cake meal resources.

## AUTHOR CONTRIBUTIONS

**Lei Dou:** Conceptualization (equal); data curation (equal); formal analysis (equal); investigation (equal); methodology (equal); resources (equal); software (equal); visualization (equal); writing – original draft (equal). **Zimu Zhang:** Data curation (equal); resources (equal). **Wenqing Yang:** Data curation (equal). **Yaobing Chen:** Data curation (equal). **Kai Luo:** Funding acquisition (equal); project administration (equal); writing – review and editing (equal). **Jianquan Kan:** Writing – review and editing (equal).

## CONFLICT OF INTEREST STATEMENT

The authors declare no conflicts of interest.

## Data Availability

All original data of this study can be obtained by contacting the corresponding author.

## References

[fsn34325-bib-0001] Ahmed, A. S. , El‐Bassiony, T. , Elmalt, L. M. , & Ibrahim, H. R. (2015). Identification of potent antioxidant bioactive peptides from goat milk proteins. Food Research International, 74, 80–88. 10.1016/j.foodres.2015.04.032 28412006

[fsn34325-bib-0002] Boengler, K. , Hilfiker‐Kleiner, D. , Heusch, G. , & Schulz, R. (2010). Inhibition of permeability transition pore opening by mitochondrial STAT3 and its role in myocardial ischemia/reperfusion. Basic Research in Cardiology, 105(6), 771–785. 10.1007/s00395-010-0124-1 20960209 PMC2978889

[fsn34325-bib-0003] Cai, Y. , Yuan, L. , Wang, K. , Liu, Q. , Xing, H. , Zhong, P. , Lin, J. , Liang, Y. , Chen, G. , Li, W. , Chen, J. , & Li, X. (2024). Eriodictyol downregulates UBA52 to promote autophagy and upregulates Nrf2/HO‐1 to inhibit oxidative stress to ameliorate non‐alcoholic fatty liver disease. Journal of Functional Foods, 113, 106041. 10.1016/j.jff.2024.106041

[fsn34325-bib-0004] Carrasco‐Castilla, J. , Hernández‐Álvarez, A. J. , Jiménez‐Martínez, C. , Gutiérrez‐López, G. F. , & Dávila‐Ortiz, G. (2012). Use of proteomics and peptidomics methods in food bioactive peptide science and engineering. Food Engineering Reviews, 4(4), 224–243. 10.1007/s12393-012-9058-8

[fsn34325-bib-0005] Chaudhary, P. , Janmeda, P. , Docea, A. O. , Yeskaliyeva, B. , Abdull Razis, A. F. , Modu, B. , Calina, D. , & Sharifi‐Rad, J. (2023). Oxidative stress, free radicals and antioxidants: Potential crosstalk in the pathophysiology of human diseases. Frontiers in Chemistry, 11, 1158198. 10.3389/fchem.2023.1158198 37234200 PMC10206224

[fsn34325-bib-0006] Chen, N. , Yang, H. , Sun, Y. , Niu, J. , & Liu, S. (2012). Purification and identification of antioxidant peptides from walnut (*Juglans regia* L.) protein hydrolysates. Peptides, 38(2), 344–349. 10.1016/j.peptides.2012.09.017 23022588

[fsn34325-bib-0007] Coelho Ferraz, A. , Bueno da Silva Menegatto, M. , Lameira Souza Lima, R. , Samuel Ola‐Olub, O. , Caldeira Costa, D. , Carlos de Magalhães, J. , Maurício Rezende, I. , Desiree LaBeaud, A. , P Monath, T. , Augusto Alves, P. , Teixeira de Carvalho, A. , Assis Martins‐Filho, O. , P Drumond, B. , & Magalhães, C. L. B. (2024). Yellow fever virus infection in human hepatocyte cells triggers an imbalance in redox homeostasis with increased reactive oxygen species production, oxidative stress, and decreased antioxidant enzymes. Free Radical Biology and Medicine, 213, 266–273. 10.1016/j.freeradbiomed.2024.01.042 38278309 PMC10911966

[fsn34325-bib-0008] Habinshuti, I. , Mu, T.‐H. , & Zhang, M. (2020). Ultrasound microwave‐assisted enzymatic production and characterisation of antioxidant peptides from sweet potato protein. Ultrasonics Sonochemistry, 69, 105262. 10.1016/j.ultsonch.2020.105262 32707458

[fsn34325-bib-0009] He, L. , He, T. , Farrar, S. , Ji, L. , Liu, T. , & Ma, X. (2017). Antioxidants maintain cellular redox homeostasis by elimination of reactive oxygen species. Cellular Physiology and Biochemistry, 44(2), 532–553. 10.1159/000485089 29145191

[fsn34325-bib-0010] Huang, L. , Zeng, Y. , Li, F. , Zheng, X. , Rao, Q. , Gajendran, B. , Varier, K. M. , Peng, T. , & Tang, L. (2023). Polyphenolic compounds from *Idesia polycarpa* Maxim. fruits ameliorate non‐alcoholic fatty liver disease by modulating lipid metabolism in oleic acid‐induced HepG2 cells and high‐fat diet‐induced mice. Journal of Functional Foods, 108, 105715. 10.1016/j.jff.2023.105715

[fsn34325-bib-0011] Igbokwe, C. J. , Feng, Y. , Louis, H. , Benjamin, I. , Quaisie, J. , Duan, Y. , Tuly, J. A. , Cai, M. , & Zhang, H. (2024). Novel antioxidant peptides identified from coix seed by molecular docking, quantum chemical calculations and invitro study in HepG2 cells. Food Chemistry, 440, 138234. 10.1016/j.foodchem.2023.138234 38145582

[fsn34325-bib-0012] Kaur, S. , Rubal , Kaur, S. , Kaur, A. , Kaur, S. , Gupta, S. , Mittal, S. , & Dhiman, M. (2023). A cross‐sectional study to correlate antioxidant enzymes, oxidative stress and inflammation with prevalence of hypertension. Life Sciences, 313, 121134. 10.1016/j.lfs.2022.121134 36544300

[fsn34325-bib-0013] Li, Y. , Peng, T. , Huang, L. , Zhang, S. , He, Y. , & Tang, L. (2019). The evaluation of lipids raw material resources with the fatty acid profile and morphological characteristics of *Idesia polycarpa* Maxim. var. vestita diels fruit in harvesting. Industrial Crops and Products, 129, 114–122. 10.1016/j.indcrop.2018.11.071

[fsn34325-bib-0014] Lin, L. , He, Y.‐L. , Liu, Y. , Hong, P. , Zhou, C. , Sun, S. , & Qian, Z.‐J. (2023). Comparative in silico and in vitro study of the stability and biological activity of an octapeptide from microalgae *Isochrysis zhanjiangensis* and its truncated short peptide. Food & Function, 14(8), 3659–3672. 10.1039/d3fo00129f 36967639

[fsn34325-bib-0015] Liu, H. , Sun, H.‐N. , Zhang, M. , Mu, T.‐H. , & Khan, N. M. (2023). Production, identification and characterization of antioxidant peptides from potato protein by energy‐divergent and gathered ultrasound assisted enzymatic hydrolysis. Food Chemistry, 405, 134873. 10.1016/j.foodchem.2022.134873

[fsn34325-bib-0016] Lu, X. , Zhang, L. , Sun, Q. , Song, G. , & Huang, J. (2019). Extraction, identification and structure‐activity relationship of antioxidant peptides from sesame (*Sesamum indicum* L.) protein hydrolysate. Food Research International, 116, 707–716. 10.1016/j.foodres.2018.09.001 30716998

[fsn34325-bib-0017] Men, D. , Dai, J. , Yuan, Y. , Jiang, H. , Wang, X. , Wang, Y. , Tao, L. , Sheng, J. , & Tian, Y. (2024). Exploration of anti‐osteoporotic peptides from *Moringa oleifera* leaf proteins by network pharmacology, molecular docking, molecular dynamics and cellular assay analyses. Journal of Functional Foods, 116, 106144. 10.1016/j.jff.2024.106144

[fsn34325-bib-0018] Mohan, C. D. , Shanmugam, M. K. , Gowda, S. G. S. , Chinnathambi, A. , Rangappa, K. S. , & Sethi, G. (2024). c‐MET pathway in human malignancies and its targeting by natural compounds for cancer therapy. Phytomedicine, 128, 155379. 10.1016/j.phymed.2024.155379 38503157

[fsn34325-bib-0019] Najafian, L. , & Babji, A. S. (2015). Isolation, purification and identification of three novel antioxidative peptides from patin (*Pangasius sutchi*) myofibrillar protein hydrolysates. LWT ‐ Food Science and Technology, 60(1), 452–461. 10.1016/j.lwt.2014.07.046

[fsn34325-bib-0020] Nathan, C. , & Cunningham‐Bussel, A. (2013). Beyond oxidative stress: An immunologist's guide to reactive oxygen species. Nature Reviews Immunology, 13(5), 349–361. 10.1038/nri3423 PMC4250048 23618831

[fsn34325-bib-0021] Nimalaratne, C. , Bandara, N. , & Wu, J. (2015). Purification and characterization of antioxidant peptides from enzymatically hydrolyzed chicken egg white. Food Chemistry, 188, 467–472. 10.1016/j.foodchem.2015.05.014 26041219

[fsn34325-bib-0022] Poprac, P. , Jomova, K. , Simunkova, M. , Kollar, V. , Rhodes, C. J. , & Valko, M. (2017). Targeting free radicals in oxidative stress‐related human diseases. Trends in Pharmacological Sciences, 38(7), 592–607. 10.1016/j.tips.2017.04.005 28551354

[fsn34325-bib-0023] Ren, L.‐K. , Fan, J. , Yang, Y. , Liu, X.‐F. , Wang, B. , Bian, X. , Wang, D. F. , Xu, Y. , Liu, B. X. , Zhu, P. Y. , & Zhang, N. (2023). Identification, in silico selection, and mechanism study of novel antioxidant peptides derived from the rice bran protein hydrolysates. Food Chemistry, 408, 135230. 10.1016/j.foodchem.2022.135230 36549163

[fsn34325-bib-0024] Sarmadi, B. H. , & Ismail, A. (2010). Antioxidative peptides from food proteins: A review. Peptides, 31(10), 1949–1956. 10.1016/j.peptides.2010.06.020 20600423

[fsn34325-bib-0025] Singh, S. , Lathoria, K. , Umdor, S. B. , Singh, J. , Suri, V. , & Sen, E. (2024). A gain of function mutation in AKT1 increases hexokinase 2 and diminishes oxidative stress in meningioma. Cytokine, 176, 156535. 10.1016/j.cyto.2024.156535 38325141

[fsn34325-bib-0026] Sun, X. , & Udenigwe, C. C. (2020). Chemistry and biofunctional significance of bioactive peptide interactions with food and gut components. Journal of Agricultural and Food Chemistry, 68(46), 12972–12977. 10.1021/acs.jafc.9b07559 31994880

[fsn34325-bib-0027] Tang, C. , Pan, J. , Li, H. , He, B. , Hong, L. , Teng, X. , & Li, D. (2019). Cyclosporin A protects trophoblasts from H_2_O_2_‐induced oxidative injury via FAK‐Src pathway. Biochemical and Biophysical Research Communications, 518(3), 423–429. 10.1016/j.bbrc.2019.07.118 31445706

[fsn34325-bib-0028] Udenigwe, C. C. , Lu, Y.‐L. , Han, C.‐H. , Hou, W.‐C. , & Aluko, R. E. (2009). Flaxseed protein‐derived peptide fractions: Antioxidant properties and inhibition of lipopolysaccharide‐induced nitric oxide production in murine macrophages. Food Chemistry, 116(1), 277–284. 10.1016/j.foodchem.2009.02.046

[fsn34325-bib-0029] Wang, L. , Ma, M. , Yu, Z. , & Du, S.‐K. (2021). Preparation and identification of antioxidant peptides from cottonseed proteins. Food Chemistry, 352, 129399. 10.1016/j.foodchem.2021.129399 33662918

[fsn34325-bib-0030] Wang, W.‐T. , Fan, M.‐L. , Hu, J.‐N. , Sha, J.‐Y. , Zhang, H. , Wang, Z. , Zhang, J.‐J. , Wang, S.‐H. , Zheng, S.‐W. , & Li, W. (2022). Maltol, a naturally occurring flavor enhancer, ameliorates cisplatin‐induced apoptosis by inhibiting NLRP3 inflammasome activation by modulating ROS‐mediated oxidative stress. Journal of Functional Foods, 94, 105127. 10.1016/j.jff.2022.105127

[fsn34325-bib-0031] Wang, X. , Fu, J. , Bhullar, K. S. , Chen, B. , Liu, H. , Zhang, Y. , Wang, C. , Liu, C. , Su, D. , Ma, X. , & Qiao, Y. (2024). Identification, in silico selection, and mechanistic investigation of antioxidant peptides from corn gluten meal hydrolysate. Food Chemistry, 446, 138777. 10.1016/j.foodchem.2024.138777 38402763

[fsn34325-bib-0032] Wen, C. , Zhang, J. , Zhang, H. , Duan, Y. , & Ma, H. (2020). Plant protein‐derived antioxidant peptides: Isolation, identification, mechanism of action and application in food systems: A review. Trends in Food Science & Technology, 105, 308–322. 10.1016/j.tifs.2020.09.019

[fsn34325-bib-0033] Wen, C. , Zhang, J. , Zhang, H. , Duan, Y. , & Ma, H. (2021). Study on the structure–activity relationship of watermelon seed antioxidant peptides by using molecular simulations. Food Chemistry, 364, 130432. 10.1016/j.foodchem.2021.130432 34182364

[fsn34325-bib-0034] Xie, M. , Ma, Y. , An, F. , Yu, M. , Zhang, L. , Tao, X. , Pan, G. , Liu, Q. , Wu, J. , & Wu, R. (2024). Ultrasound‐assisted fermentation for antioxidant peptides preparation from okara: Optimization, stability, and functional analyses. Food Chemistry, 439, 138078. 10.1016/j.foodchem.2023.138078 38086234

[fsn34325-bib-0035] Yang, W. , Zhang, Z. , Chen, Y. , & Luo, K. (2023). Evaluation of the use of *Idesia polycarpa* Maxim protein coating to extend the shelf life of European sweet cherries. Frontiers in Nutrition, 10, 1283086. 10.3389/fnut.2023.1283086 38045816 PMC10693450

[fsn34325-bib-0036] Zhang, L. , Xu, L. Y. , Tang, F. , Liu, D. , Zhao, X. L. , Zhang, J. N. , Xia, J. , Wu, J.‐J. , Yang, Y. , Peng, C. , & Ao, H. (2024). New perspectives on the therapeutic potential of quercetin in non‐communicable diseases: Targeting Nrf2 to counteract oxidative stress and inflammation. Journal of Pharmaceutical Analysis, 14, 100930. 10.1016/j.jpha.2023.12.020 39005843 PMC11245930

[fsn34325-bib-0037] Zhang, Q. , Yu, Z. , & Zhao, W. (2023). Identification and action mechanism of novel antioxidative peptides from copra meal protein. LWT ‐ Food Science and Technology, 188, 115425. 10.1016/j.lwt.2023.115425

[fsn34325-bib-0038] Zhang, X. , Li, W. , Yu, G. , Zuo, X. , Luo, W. , Zhang, J. , Tan, B. , Fu, A. , & Zhang, S. (2020). Evaluation of *Idesia polycarpa* Maxim fruits extract as a natural green corrosion inhibitor for copper in 0.5 M sulfuric acid solution. Journal of Molecular Liquids, 318, 114080. 10.1016/j.molliq.2020.114080

[fsn34325-bib-0039] Zheng, X. , Chi, H. , Ma, S. , Zhao, L. , & Cai, S. (2023). Identification of novel α‐glucosidase inhibitory peptides in rice wine and their antioxidant activities using in silico and in vitro analyses. LWT ‐ Food Science and Technology, 178, 114629. 10.1016/j.lwt.2023.114629

[fsn34325-bib-0040] Zhou, N. , Zhong, Y. , & Liu, H. (2024). Characterization and relationship analysis of antioxidant and anti‐inflammatory peptides in pomelo fruitlet albumin. Food Chemistry, 446, 138798. 10.1016/j.foodchem.2024.138798 38452501

